# Comparative effectiveness and safety of GATT with or without ABiC in patients with open-angle glaucoma

**DOI:** 10.3389/fmed.2025.1581608

**Published:** 2025-08-26

**Authors:** Weijia Zhang, Yiwei Wang, Xin Chen, Ke Zhang, Zhen Yang, Jinghong Sang, Chun Zhang, Huaizhou Wang

**Affiliations:** ^1^Department of Ophthalmology, Peking University Third Hospital, Beijing, China; ^2^Department of Ophthalmology, Henan Provincial People’s Hospital, Zhengzhou, China; ^3^Beijing Tongren Eye Center, Beijing Tongren Hospital, Capital Medical University, Beijing, China; ^4^Beijing Ophthalmology and Visual Sciences Key Laboratory, Beijing, China; ^5^Beijing Tsinghua Changgung Hospital Eye Center, School of Clinical Medicine, Tsinghua Medicine, Tsinghua University, Beijing, China; ^6^Beijing Visual Science and Translational Eye Research Institute (BERI), Beijing, China

**Keywords:** gonioscopy-assisted transluminal trabeculotomy, ab interno canaloplasty, minimally invasive glaucoma surgery, open-angle glaucoma, intraocular pressure

## Abstract

**Background:**

This is the first comprehensive comparison between gonioscopy-assisted transluminal trabeculotomy (GATT) and GATT combined with ab interno canaloplasty (ABiC) in patients with OAG.

**Purpose:**

The purpose of this study was to compare the efficacy and safety of gonioscopy-assisted transluminal trabeculotomy (GATT) combined with ab interno canaloplasty (ABiC) with those of GATT alone in patients with open-angle glaucoma (OAG).

**Design:**

This was a retrospective, comparative case series.

**Participants:**

Patients with primary open angle glaucoma who underwent GATT (27 eyes in Group 1) or GATT + ABiC (26 eyes in Group 2) were included.

**Methods:**

Outcomes including intraocular pressure (IOP), glaucoma medications, and surgical complications were analyzed.

**Main outcome measures:**

Surgical success was defined in terms of IOP and medication use as follows: (1) a preoperative IOP > 21 mmHg and a postoperative IOP ≤ 21 mmHg with at least a 20% reduction from baseline with (qualified success) or without (complete success) glaucoma medications or (2) a preoperative IOP < 21 mmHg while taking 3 or more glaucoma medications and a postoperative IOP ≤ 21 mmHg with a reduction of more than two medications (qualified success) or with no medications (complete success).

**Results:**

At 12 months, the mean IOP was 14.8 ± 2.2 mmHg in Group 1 and 16.6 ± 2.3 mmHg in Group 2 (*p* = 0.008). The number of medications was 0.6 ± 1.0 in Group 1 and 0.9 ± 1.3 in Group 2 (*p* = 0.334). At 24 months, the mean IOP was 15.3 ± 2.0 mmHg in Group 1 and 15.5 ± 2.4 mmHg in Group 2 (*p* = 0.676). The number of medications was 0.5 ± 0.9 in Group 1 and 0.9 ± 1.1 in Group 2 (*p* = 0.197). The complete success rates were 63.0% in Group 1 and 50.0% in Group 2 (*p* = 0.16), and the qualified success rates were 81.5% in Group 1 and 76.9% in Group 2 (*p* = 0.51).

**Conclusion:**

The GATT procedure, with or without ABiC, is safe and effective in decreasing the IOP and the number of antiglaucoma medications used.

## 1 Introduction

Glaucoma is a group of diseases characterized by progressive optic neuropathy and visual field loss and is the leading cause of irreversible blindness worldwide (1, 2). The specific pathogenesis of OAG is not fully understood. Elevated intraocular pressure (IOP) is the primary risk factor for the development and progression of OAG, which is caused by increased resistance to aqueous outflow. The juxtacanalicular tissue of the trabecular meshwork and inner wall of Schlemm’s canal is thought to be the site of greatest resistance to aqueous outflow (3, 4). Therefore, procedures aimed at facilitating physiologic aqueous outflow include removing, disrupting, or bypassing the trabecular meshwork and inner wall of Schlemm’s canal.

The only known modifiable risk factor for OAG is IOP; therefore, lowering IOP is the aim of medical and surgical treatments for glaucoma (5). In the last two decades, minimally invasive or microinvasive glaucoma surgery (MIGS) has been increasingly performed (6). MIGS represents a relatively new category of procedures that lower IOP with limited or no disruption to the conjunctiva or sclera and has thus become the standard of care for glaucoma (7, 8). Several canal-based procedures aimed at restoring physiological outflow through the trabecular pathway, including canaloplasty and circumferential trabeculotomy, have been developed.

Gonioscopy-assisted transluminal trabeculotomy (GATT) is a modification of trabeculotomy and is considered MIGS owing to the use of an ab interno approach to avoid the disadvantages of ab externo trabeculotomy (9). GATT has since shown promising results in the treatment of most types of glaucoma, including OAG (10). In various retrospective studies, researchers reported the outcomes of GATT and ABiC for treating different types of glaucoma (11, 12). The success rate was relatively high in various studies.

Ab interno canaloplasty (ABiC) is a MIGS aimed at addressing all aspects of aqueous outflow (13) and is characterized by a good safety profile, association with rapid rehabilitation, ab interno microincision, correlation with minimal trauma, and moderate pressure-lowering effectiveness (14). For patients with OAG, ABiC has been proven safe and effective in lowering IOP and reducing the use of hypotensive medications (15). ABiC is considered advantageous owing to its theoretical appeal (16, 17). Active aqueous pumps and IOP stabilizers are used to preserve the function of the trabecular meshwork in ABiC (18).

Although the processes of both ABiC and GATT are similar, the combination of GATT and ABiC may more effectively lower IOP than GATT alone. During the ABiC procedure, Healon GV is injected into the SC and collector channels. The physical expansion of the SC and CC is one of the mechanical factors of the IOP-lowering effect of ABiC. The Healon GV in the CC may prevent blood reflux into the anterior chamber and decrease the probability of hyphema. Therefore, the GATT+ABiC procedure may increase the success rate and decrease complications. This may result in a high success rate in patients undergoing treatment to lower IOP.

To date, no reports have compared the efficacy and safety of the combination of GATT with ABiC with those of GATT alone. Researchers in only one study reported the use of the combination of GATT with ABiC and phacoemulsification. The aim of this study was to compare the efficacy and safety of GATT combined with ABiC with those of GATT alone in patients with open-angle glaucoma.

## 2 Methods

### 2.1 Study design

The medical charts of patients with OAG who underwent either gonioscopy-assisted transluminal trabeculotomy (Group 1) or gonioscopy-assisted transluminal trabeculotomy combined with ab interno canaloplasty (Group 2) between Oct 2020 and May 2021 at Beijing Tongren Hospital were retrospectively reviewed. Diagnostic criteria of OAG included: open anterior chamber angle configuration on gonioscopy (Shaffer grade ≥ 3), glaucomatous optic neuropathy (neural rim thinning, focal notching, vertical cup-to-disk ratio > 0.6 or cup-to-disk ratio asymmetry ≥ 0.2) and/or glaucomatous visual field defects and a history of hypotensive medication use. Only patients with > 12 months of follow-up were included. Exclusion criteria included patients with pseudoexfoliative glaucoma, pigmentary glaucoma, glaucoma associated with ocular trauma and glaucoma associated with ocular inflammation. All the surgeries were performed by Dr. HZ Wang. The study was conducted in accordance with the tenets of the Declaration of Helsinki and approved by the Institutional Ethics Committee of Beijing Tongren Hospital. Written informed consent was obtained from all the subjects. All patients included in this study were diagnosed with OAG, had uncontrolled IOP, or exhibited progressive worsening of visual field defects despite treatment with medication or were intolerant to glaucoma medications. All patients underwent comprehensive ophthalmic examinations preoperatively, which included gonioscopy of the anterior chamber angle. The angle of the anterior chamber and IOP were assessed during follow-up visits (1 month, 3 months, 6 months, 12 months, 18 months, and 24 months), and antiglaucoma medication usage was recorded before surgery and at each follow-up visit. Intraoperative and postoperative complications were also documented and analyzed. Combination glaucoma medications were evaluated according to the number of active agents in the medication. An IOP ≥ 30 mmHg within 1 month of surgery was defined as an IOP spike (19).

### 2.2 Surgical procedure and postoperative care

All the surgeries were performed by a single surgeon (HZ Wang) using an illuminated ophthalmic microcatheter (iTrack 250A; iScience Interventional, Menlo Park, CA) for GATT and Healon GV (Abbott Laboratories, Chicago, IL, United States) for ABiC. The steps of the GATT procedure have been reported previously (9). A clear corneal incision was made at the temporal site. Viscoelastic material was injected into the anterior chamber. A paracentesis track, oriented tangentially, was then placed in the superonasal or inferonasal quadrant, serving as the entry site for the microcatheter. The microscope and the patient’s head were then oriented to facilitate the best visualization of the nasal angle by the surgeon via a Swan–Jacob gonio lens. A 1 mm incision was made at the nasal angle for goniotomy, and then circumferential catheterization and trabeculotomy were performed. If the microcatheter stopped somewhere in Schlemm’s canal, ab interno trabeculotomy was performed, and the microcatheter was then placed in the opposite direction to excise the canal and achieve as close to a 360-degree opening as possible. The viscoelastic substance was gently removed, and the corneal wounds were hydrated to ensure watertight closure.

The steps of GATT combined with ABiC were the same as those of GATT alone. After the illuminated microcatheter was passed circumferentially 360° through the goniotomy site via microsurgical forceps, the microcatheter was pulled out with the forceps, and Healon GV was injected simultaneously (2 clips for each hour on the clock), the amount of Healon GV can be tailored on the eye, but it is an average of 100 μL over the entirety of Schlemm’s canal. After the entire canal was cannulated, the microcatheter was placed into the SC again, and circumferential trabeculotomy was performed by pulling both ends of the microcatheter. Afterward, the procedure was the same as that for GATT.

Postoperatively, tobramycin–dexamethasone (TobraDex, Alcon, Rijksweg, Belgium) and pranoprofen (Pranopulin, Senju Pharmaceutical, Osaka, Japan) eye drops were used 4 times daily for 2–4 weeks. Pilocarpine 2% (Bausch & Lomb, Rochester, NY, United States) was used 4 times daily for 2 months to prevent peripheral anterior synechia regardless of the IOP and was therefore not considered antiglaucoma medication during this time. IOP elevation within the first month after surgery was not treated unless the IOP exceeded 25 mmHg. Prostaglandins and medications that suppress aqueous humor formation were preferentially prescribed. The anterior chamber was irrigated in cases of massive hyphema (blood filling one half or more of the anterior chamber) or increased IOP.

### 2.3 Outcome measures and statistical analysis

The surgical outcomes included reductions in IOP and glaucoma medication use at the 1-, 3-, 6-, 12-, 18-, and 24-month follow-up visits, as well as the rates of surgical success and complications. Surgical success was characterized as follows: (1) a preoperative IOP > 21 mmHg and a postoperative IOP ≤ 21 mmHg with at least a 20% reduction from baseline with (qualified success) or without (complete success) glaucoma medication use or (2) a preoperative IOP < 21 mmHg while being treated with 3 or more glaucoma medications and a postoperative IOP ≤ 21 mmHg with a reduction of more than two medications (qualified success) or with no glaucoma medications (complete success). Based on preoperative IOP and medications, two subgroups based on preoperative scenarios were characterized: IOP > 21 mmHg (subgroup A) and IOP < 21 mmHg on ≥3 medications (subgroup B).

Categorical variables (sex, side of the eye, diagnosis by eye, postoperative IOP, IOP reduction, and medication count) were tested via the chi-square test or Fisher’s exact test, as appropriate. The Mann–Whitney U test was performed for prior glaucoma surgery, preoperative IOP, preoperative glaucoma medications, follow-up duration, mean percent reduction in IOP and medication reduction between groups. Multivariate logistic regression was performed for preoperative IOP, medication number, prior surgery, and cup-disk ratio to address potential confounders. Repeated measures variables were analyzed via mixed-effects models, with the Bonferroni-corrected significance level used for multiple comparisons. Independent samples *t* tests were used to compare age and absolute IOP reduction between groups. The cumulative probability of success was analyzed via Kaplan–Meier survival analysis with the log rank test. Chi-square test were used for intergroup comparison. A *p* value < 0.05 (2-tailed) indicated statistical significance.

## 3 Results

### 3.1 Demographics and ocular characteristics

Data analysis was performed for 53 eyes of 48 patients. The demographics and ocular characteristics are summarized in Table 1. A total of 27 eyes from 25 patients who underwent GATT (Group 1) and 26 eyes from 23 patients who underwent GATT combined with ABiC (Group 2) were included, and all patients were diagnosed with OAG. The two groups were comparable in terms of sex (*p* = 0.951), age (*p* = 0.785), right eye rate (*p* = 0.341), prior surgery (*p* = 0.125), preoperative IOP (*p* = 0.376), number of preoperative glaucoma medications (*p* = 0.428), C/D (*p* = 0.272), and follow-up duration (*p* = 0.376).

**Table 1:** Demographic and ocular characteristics.

Parameters	GATT group	GATT + ABiC group	*P* value
No. of patients	25	23	–
Sex (male/female)	15/10	14/9	0.951*
No. of eyes	27	26	–
OD/OS	10/17	13/13	0.341*
Age (years, mean ± SD)	36.8 ± 11.1	37.8 ± 14.1	0.785^§^
Surgery in phakic eyes	27 (100%)	26 (100%)	–
Prior surgery, median (range)	0 (0–2)	0 (0–2)	0.125^ǂ^
Median follow-up duration in months (range)	24.9 (12–33)	24.3 (12–30)	0.536^ǂ^
Preoperative IOP, mean ± SD (mmHg)	27.1 ± 11.1	24.5 ± 8.4	0.376^ǂ^
Preoperative no. of medications, mean ± SD	3.5 ± 1.0	3.7 ± 0.8	0.428^ǂ^
C/D, mean ± SD	0.9 ± 0.1	0.8 ± 0.1	0.272^§^

^*^Chi-square test.

^§^*t* test.

^ǂ^Mann–Whitney U test.

GATT, gonioscopy-assisted transluminal trabeculotomy; ABiC, ab interno canaloplasty; OD, oculus dexter; OS, oculus sinister; SD, standard deviation; POAG, primary open-angle glaucoma; JOAG, juvenile open-angle glaucoma; SOAG, secondary open-angle glaucoma; IOP, intraocular pressure; C/D, cup–disk ratio.

### 3.2 Intraocular pressure

**Figure 1:**
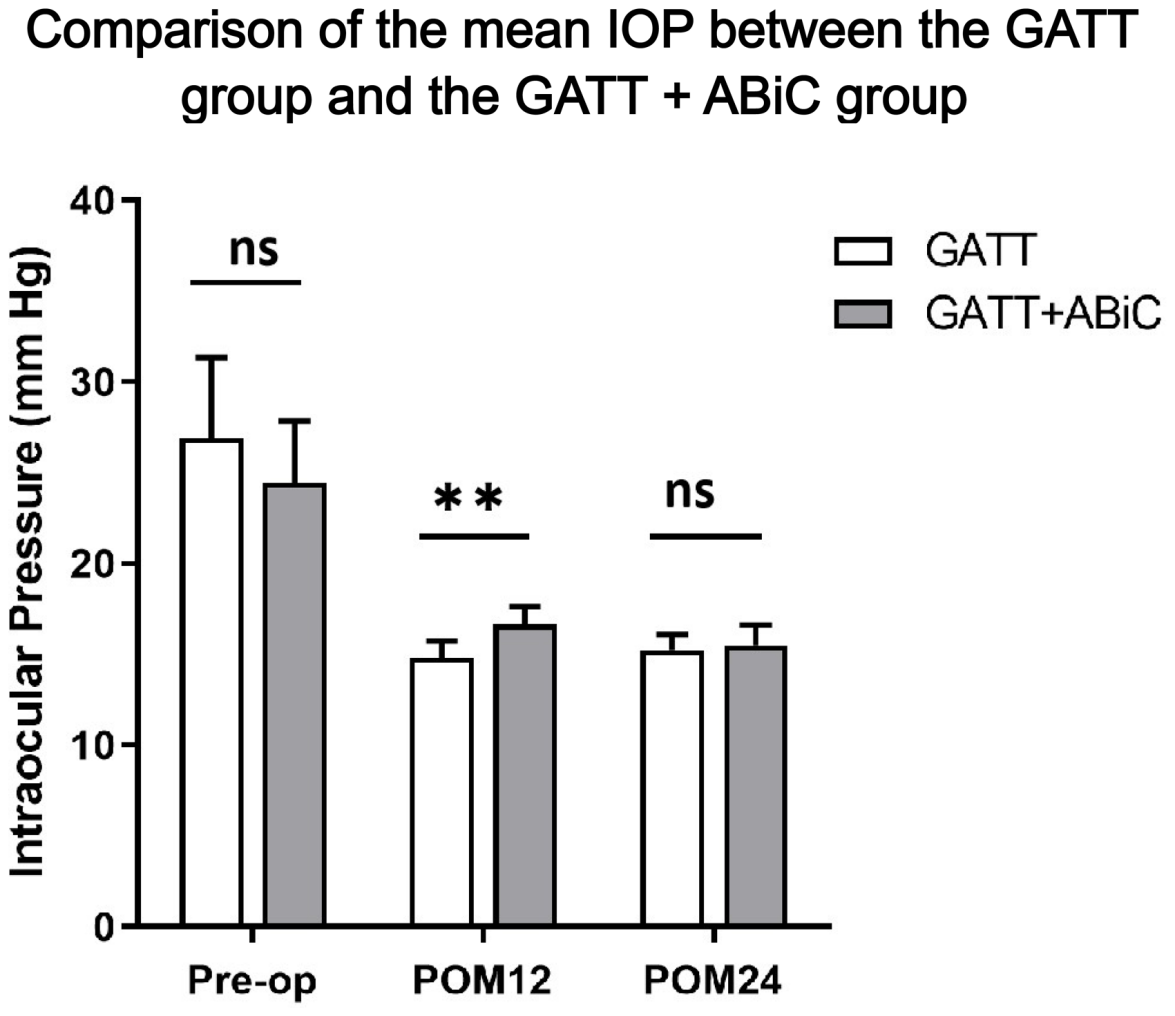
Comparison of the mean IOP between the GATT group and the GATT + ABiC group. Error bars show standard error to the 95% CIs. Preop, preoperative; POM, postoperative month; IOP, intraocular pressure; GATT, gonioscopy-assisted transluminal trabeculotomy; ABiC, ab interno canaloplasty.

**Figure 2:**
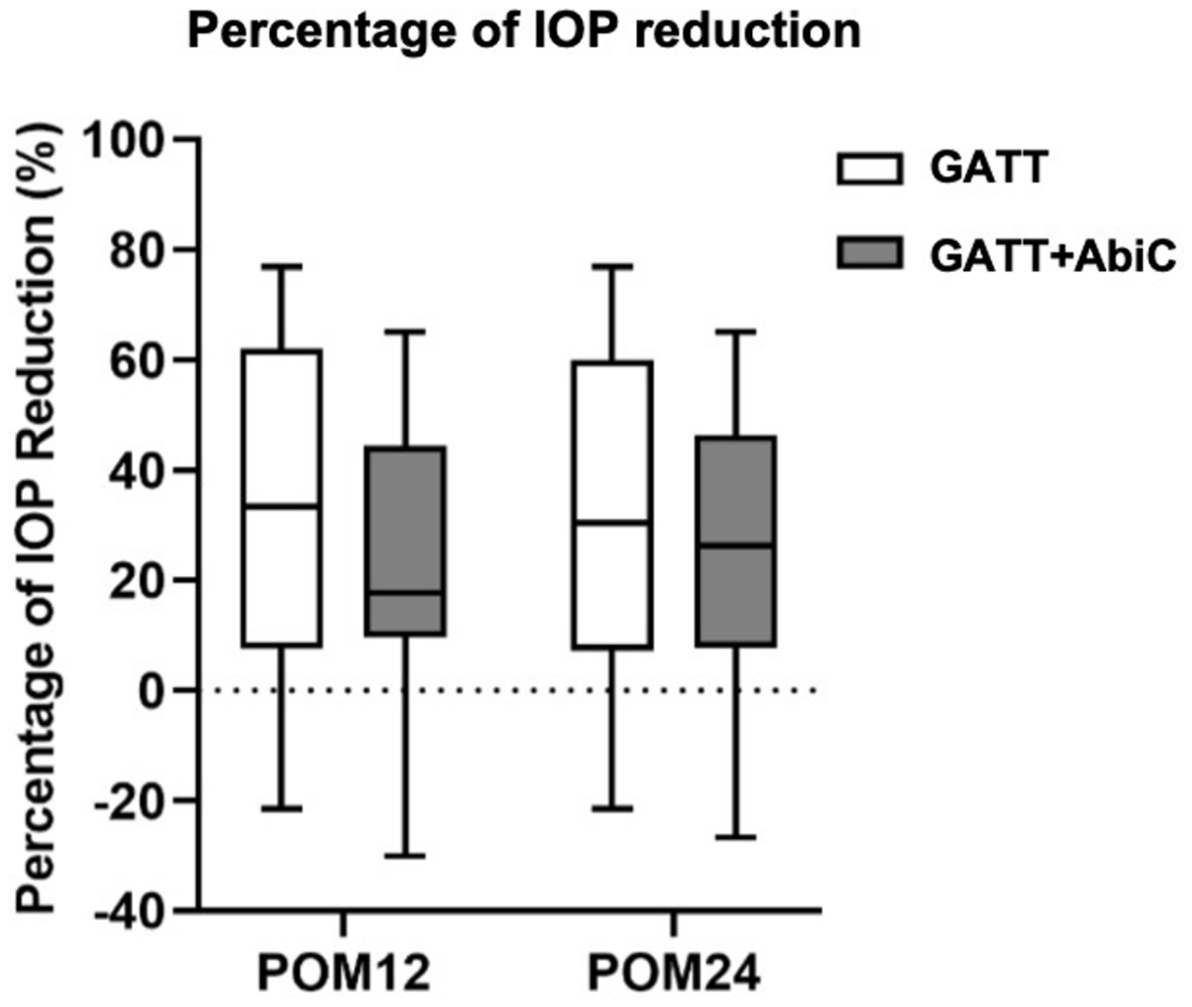
Percentage of IOP reduction. POM, postoperative month; IOP, intraocular pressure; GATT, gonioscopy-assisted transluminal trabeculotomy; ABiC, ab interno canaloplasty.

Table 2 shows the results for IOP and glaucoma medication use. The mean IOP decreased from 27.1 ± 11.1 mmHg in Group 1 and 24.5 ± 8.4 mmHg in Group 2 preoperatively to 14.8 ± 2.2 mmHg in Group 1 and 16.6 ± 2.3 mmHg in Group 2 at 12 months postoperatively and to 15.3 ± 2.0 mmHg in Group 1 and 15.5 ± 2.4 mmHg in Group 2 at 24 months postoperatively. Both procedures significantly reduced IOP, as measured at the follow-up visits (*p* < 0.001 for each postoperative visit compared with the baseline) (Figure 1). Throughout the 12-month follow-up, the IOP was lower in Group 1 than in Group 2 (*p* = 0.008), but there was no significant difference in the mean IOP between the two groups in the first month (POM) 24 (*p* = 0.676, Figure 2).

**Table 2:** IOP and medication usage in the GATT group and the GATT+ABiC group.

Parameters	POM 12			POM 24		
	GATT	GATT+ABiC	*P* value	GATT	GATT+ABiC	*P* value
No. of subjects (eyes)	23 (25)	21 (23)		22 (24)	19 (21)	
IOP (mmHg, mean ± SD)	14.8 ± 2.2	16.6 ± 2.3	0.008^§^	15.3 ± 2.0	15.5 ± 2.4	0.676^§^
IOP distribution	No. of eyes (%)			No. of eyes (%)		
IOP ≤ 15	14 (56.0)	9 (39.1)	0.243^*^	12 (50.0)	12 (57.1)	0.632^*^
IOP ≤ 18	24 (96.0)	17 (73.9)	0.044^*^	24 (100)	17 (81.0)	0.040^*^
IOP ≤ 21	25 (100.0)	22 (95.7)	0.479^*^	24 (100)	21 (100)	–
IOP > 21	0 (0)	1 (4.3)	–	0 (0)	0 (0)	–
IOP reduction (from baseline)	33.56%	23.73%	0.221^§^	30.13%	26.15%	0.633^§^
IOP reduction < 20%	10 (40.0%)	12 (52.2%)	0.398*	10 (41.7%)	7 (33.3%)	0.565*
IOP reduction ≥ 20%	15 (60.0%)	11(47.8%)	–	14 (58.3%)	14 (66.7%)	–
No. of medications (mean ± SD)	0.6 ± 1.0	0.9 ± 1.3	0.334^ǂ^	0.5 ± 0.9	0.9 ± 1.1	0.197^ǂ^
Medication count	No. of eyes (%)			No. of eyes (%)		
0	16 (64.0)	12 (51.2)	0.406^*^	16 (66.7)	10 (47.6)	0.197^*^
≤1	22 (88.0)	18 (78.3)	0.454^*^	21 (87.5)	16 (76.2)	0.443^*^
≤2	24 (96.0)	20 (87.0)	0.338^*^	22 (91.7)	19 (90.5)	1.000^*^
≤3	24 (96.0)	21 (91.3)	0.601^*^	24 (100.0)	20 (95.2)	0.467^*^
≤4	25 (100.0)	23 (100.0)	–	24 (100.0)	21 (100.0)	–

^§^*t* test.

^ǂ^Mann–Whitney U test.

*Chi-square test or Fisher’s exact test.

POM, postoperative month; GATT, gonioscopy-assisted transluminal trabeculotomy; ABiC, ab interno canaloplasty; IOP, intraocular pressure; SD, standard deviation.

At the 12-month follow-up, the mean IOP was 14.8 ± 2.2 mmHg in Group 1; 14 eyes (56.0%) had an IOP ≤ 15 mmHg, 24 eyes (96.0%) had an IOP ≤ 18 mmHg, and 25 eyes (100.0%) had an IOP ≤ 21 mmHg. In Group 2, the mean IOP was 16.6 ± 2.3 mmHg, of which 9 eyes (39.1%) had an IOP ≤ 15 mmHg, 17 eyes (73.9%) had an IOP ≤ 18 mmHg, and 22 eyes (95.7%) had an IOP ≤ 21 mmHg. Only 1 eye had an IOP > 21 mmHg (4.3%). The percentages of IOP reduction were 33.56 and 23.73%, respectively (*p* = 0.221). Overall, 60.0% of Group 1 patients and 47.8% of Group 2 patients experienced a ≥ 20% reduction in the baseline IOP (*p* = 0.398). There was no significant difference in the distribution of IOP between the two groups. At the 24-month follow-up, the mean IOP remained at 15.3 ± 2.0 mmHg in Group 1, with 12 eyes (50.0%) having an IOP ≤ 15 mmHg, 24 eyes (100%) having an IOP ≤ 18 mmHg and 24 eyes (100%) having an IOP ≤ 21 mmHg. In Group 2, the mean IOP decreased to 15.5 ± 2.4 mmHg, with 12 eyes (57.1%) having an IOP ≤ 15 mmHg, 17 eyes (81.0%) having an IOP ≤ 18 mmHg and 21 eyes (100%) having an IOP ≤ 21 mmHg. The percentages of IOP reduction were 30.13 and 26.15%, respectively (*p* = 0.633). Overall, 58.3% of Group 1 patients and 66.7% of Group 2 patients experienced a ≥ 20% reduction from the baseline IOP (*p* = 0.565). There was no significant difference in the distribution of IOP between the two groups (Figure 2).

### 3.3 Medication use

**Figure 3:**
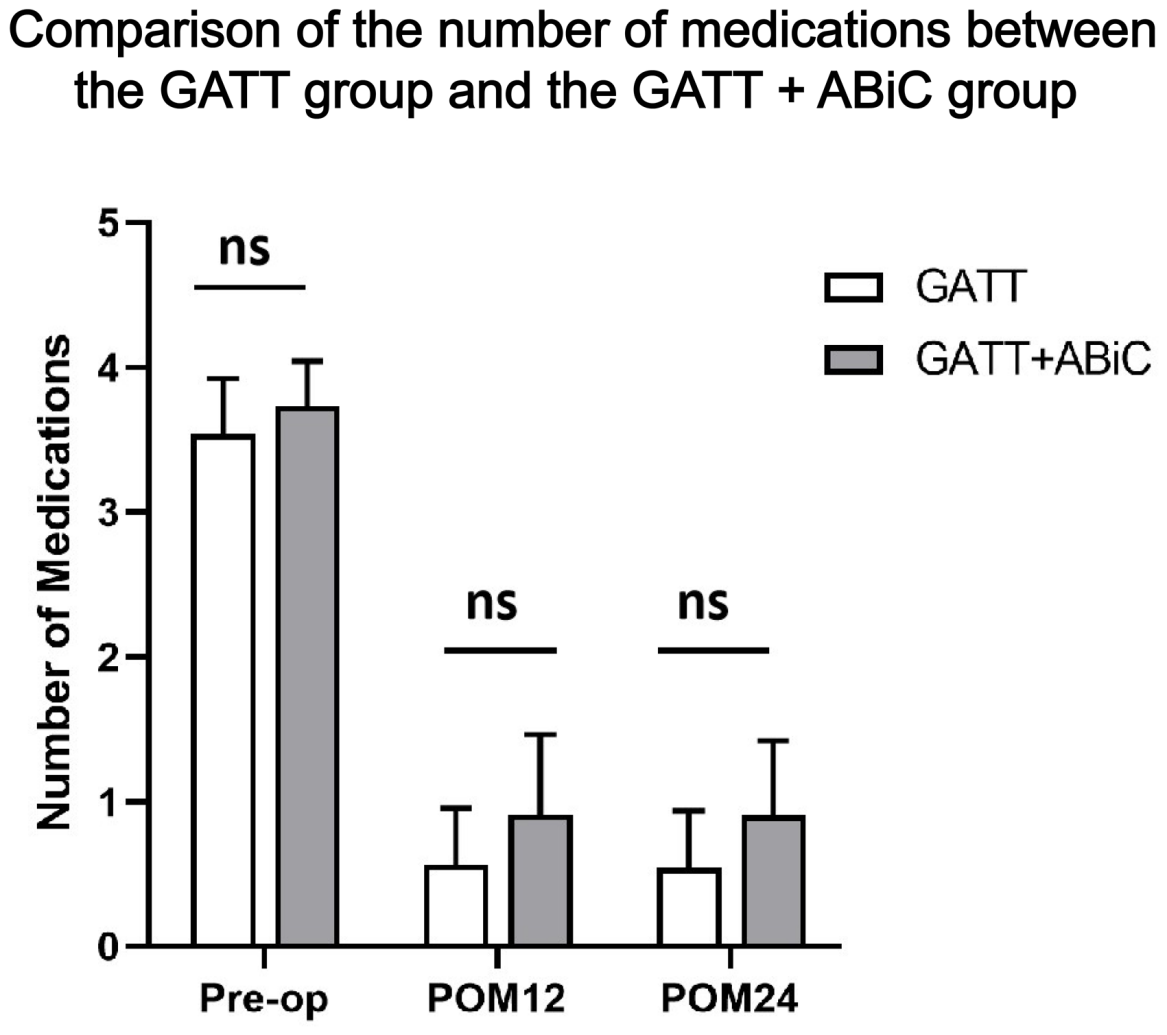
Comparison of the number of medications used between the GATT group and the GATT + ABiC group. Error bars show standard error to the 95% CIs. Preop, preoperative; POM, postoperative month; IOP, intraocular pressure; GATT, gonioscopy-assisted transluminal trabeculotomy; ABiC, ab interno canaloplasty.

**Figure 4:**
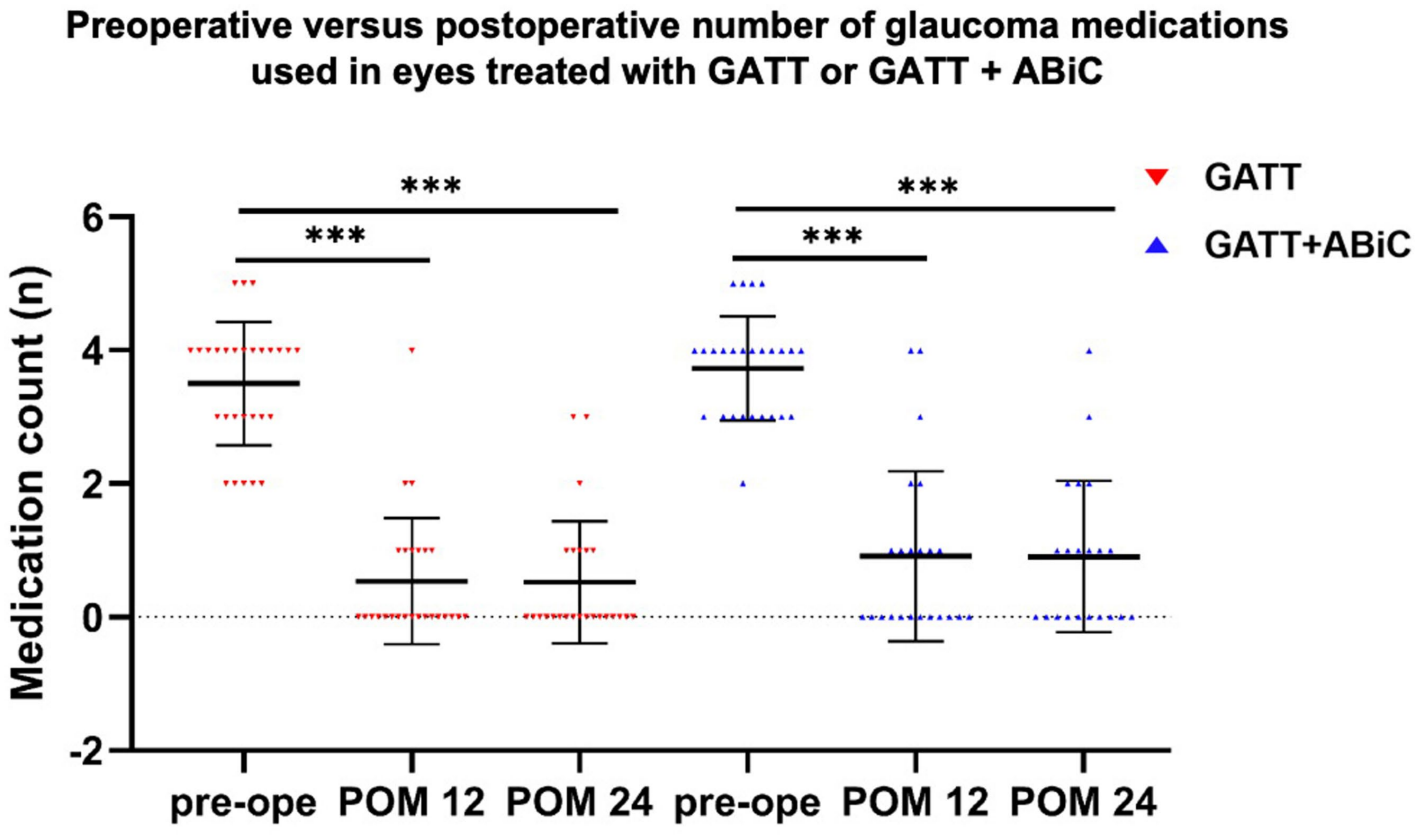
Preoperative versus postoperative number of glaucoma medications used in eyes treated with GATT or GATT combined with ABiC. Compared with that before surgery, the number of glaucoma medications used at 12 and 24 months postoperatively was significantly lower than that preoperatively (Mann–Whitney U test, *** *p* < 0.001).

The mean number of glaucoma medications decreased from 3.5 ± 1.0 preoperatively to 0.6 ± 1.0 at 12 months postoperatively and 0.5 ± 0.9 at 24 months postoperatively in Group 1, and from 3.7 ± 0.8 preoperatively to 0.9 ± 1.3 at 12 months and 0.9 ± 1.1 at 24 months postoperatively in Group 2. The mean number of glaucoma medications used was significantly lower at all postoperative time points than at the baseline (*p* < 0.001) in both groups (Figures 3, 4). There was no significant difference in the mean reduction in the use of glaucoma medications between the two groups at 12 months postoperatively (*p* = 0.334) or 24 months postoperatively (*p* = 0.197) (Table 2).

Overall, 64.0% of those in Group 1 and 51.2% of those in Group 2 were medication free 12 months postoperatively (*p* = 0.406), and 66.7% in Group 1 and 47.6% in Group 2 remained medication free (*p =* 0.197) at 24 months postoperatively. At the 12-month follow-up, 88.0% of those in Group 1 used ≤ 1 medication, 96.0% used ≤ 2 medications, 96.0% used ≤ 3 medications, and 100.0% used ≤ 4 medications. In Group 2, 78.3% of the patients used ≤ 1 medication, 87.0% used ≤ 2 medications, 91.3% used ≤ 3 medications, and 100.0% used ≤ 4 medications. There was no significant difference in the number of glaucoma medications used. At the 24-month follow-up, 87.5% of those in Group 1 used ≤ 1 type of medication, 91.7% used ≤ 2 medications, 100.0% used ≤ 3 medications, and 100.0% used ≤ 4 medications; in Group 2, 76.2% of the patients used ≤ 1 medication, 90.5% used ≤ 2 medications, 95.2% used ≤ 3 medications, and 100.0% used ≤ 4 medications. There was no significant difference in the number of glaucoma medications used (Table 2).

### 3.4 Success rate

**Figure 5:**
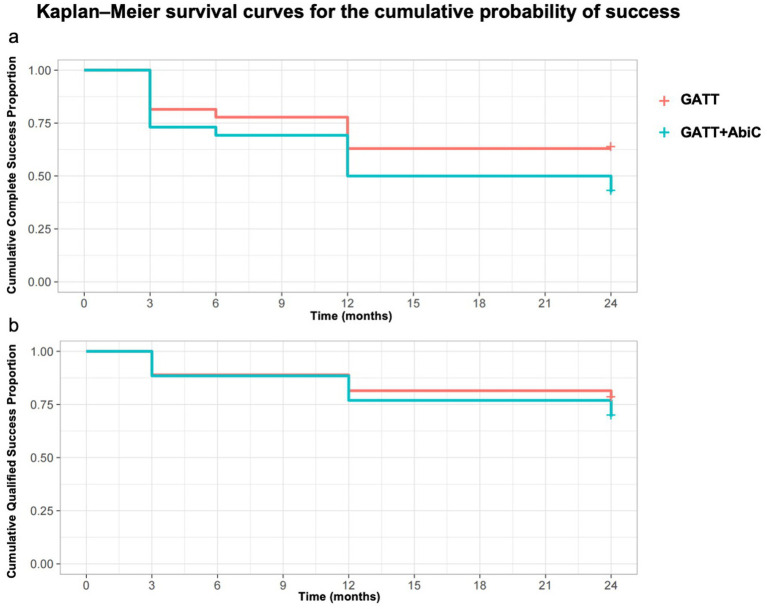
Kaplan–Meier survival curves for the cumulative probability of success. **(a)** Kaplan–Meier survival curves for the cumulative probability of complete success. **(b)** Kaplan–Meier survival curves for the cumulative probability of qualified success. GATT, gonioscopy-assisted transluminal trabeculotomy; ABiC, ab interno canaloplasty.

Figure 5 shows the results of the Kaplan–Meier survival analyses of the cumulative probabilities of success. At the 24-month follow-up visit, the complete success rates were 63.0% in Group 1 and 50.0% in Group 2 (*p* = 0.160), and the qualified success rates were 81.5% in Group 1 and 76.9% in Group 2 (*p* = 0.510). There was no significant difference between the two groups. Results of multivariate logistic regression confirmed that surgical group assignment did not significantly influence success rates (Group 1 vs. Group 2: OR = 1.12, 95% CI 0.87–1.44; *p* = 0.38).

Then we compared the success rates by different subgroups (Supplementary Table S1). Subgroup A showed higher success rates while subgroup B showed lower success rates at 24 POM in both Group 1 and Group 2. There was no statistical difference between Group 1 and Group 2 in either subgroup (*p* > 0.05).

### 3.5 Complications and subsequent interventions

The complications are summarized in Table 3. No patients in either of the two groups experienced vision-threatening complications, and complications did not differ significantly between the two groups. No complications, except for hyphema, occurred during surgery, and hyphema was also the most common postoperative complication in both groups. All patients presented with different degrees of hyphema on the first day after surgery. In most patients in both groups, the hyphema resolved spontaneously by the second week. Twelve eyes (48.0%) in Group 1 and 9 eyes (34.7%) in Group 2 experienced IOP spikes, which occurred mainly in the first postoperative week. Eyes with an IOP spike, which was probably due to massive hyphema, were successfully treated via anterior chamber irrigation. One eye in Group 2 experienced membrane detachment, and anterior chamber plasty was performed on day 25 after surgery.

**Table 3:** Postoperative complications and subsequent surgeries.

Parameters	GATT group	GATT + ABiC group	*P* value
Postoperative complications			
Hyphema	25 (100.0%)	23 (100.0%)	–
Spike in intraocular pressure	12 (48.0%)	8 (34.7%)	0.861^†^
In the first week after surgery	5 (20.0%)	3 (13.0%)	
In the second week after surgery	7 (28.0%)	5 (21.7%)	
Descemet membrane detachment	0 (0)	1 (4.3%)	1.000^§^
Subsequent surgeries			
Trabeculectomy	2 (8.0%)	1 (4.3%)	0.273^†^

^†^Chi-square test.

^§^Fisher exact test.

GATT, gonioscopy-assisted transluminal trabeculotomy; ABiC, ab interno canaloplasty; IOP, intraocular pressure; SD, standard deviation.

Two eyes in Group 1 and one eye in Group 2 underwent additional glaucoma surgery to control IOP, and all of these patients underwent filtration surgery. One patient in Group 1 underwent additional glaucoma surgery at 3 months postoperatively, and the other two patients underwent additional glaucoma surgery at one and a half years postoperatively.

## 4 Discussion

Both GATT and ABiC are safe procedures. The similarity in surgical technique between the two procedures makes it important to identify which procedure is more effective for IOP reduction and whether combined surgery is better than single surgery. The researchers in this study compared the short-term outcomes of GATT and ABiC for OAG (20). We found that both procedures significantly reduced IOP and glaucoma medication usage. At 24 months after the operation, the mean IOP was not different between the two groups; 100% of patients in Group 1 and Group 2 had an IOP ≤ 21. A total of 66.7% of the patients in Group 1 no longer needed medication, which was higher than the 47.6% reported in Group 2. The 24-month cumulative rate of complete surgical success was 63.0% in Group 1, which was higher than the 50.0% reported in Group 2, and the qualified success rates were 81.5% in Group 1 and 76.9% in Group 2. This is the first study to directly compare the efficacy of GATT with that of GATT+ABiC for IOP reduction in management of OAG. The strengths of this study are its long follow-up duration, low dropout rate, and standardized inclusion and exclusion criteria.

The effectiveness and outcomes of GATT reported in our study are consistent with findings from previous clinical studies (19, 21, 22). The effectiveness of GATT was first described by Grover et al. (9) in 2014. In their 12-month follow-up retrospective study, they reported an IOP reduction of 11.1 ± 6.1 mmHg (40%), with an average of 1.1 ± 1.8 fewer antiglaucoma medications in 57 patients who underwent GATT with or without cataract surgery for POAG. Furthermore, they reported an overall success rate of 91% in patients who underwent GATT with or without cataract surgery (22). Faria et al. (12) reported a success rate of 87% at 12 months in 73 patients with OAG who underwent GATT with or without cataract surgery, and the IOP decreased from 24.9 ± 8.5 to 12.1 ± 2.1 mmHg. Cubuk and Unsal (23) reported an overall mean decrease in intraocular pressure of 11.3 ± 9.3 mmHg at 12 months with or without cataract surgery, and the surgical success rate with medication was 91.8% (33/37). A recent study revealed that GATT is safe and effective for treating various forms of open-angle glaucoma in long-term follow-up. The mean IOP was 27.0 ± 10.0 mmHg preoperatively and was reduced to 14.8 ± 6.5 mmHg at 4 years, and the mean number of medications used decreased from 3.2 ± 1.0 preoperatively to 2.3 ± 1.0 at 4 years (24). Bektas et al. (25) reported that the surgical success rate was 81.1% for GATT surgery, and in our previous study of GATT combined with phacoemulsification surgery, the overall success rate at 24 months was 86.21% for GATT combined with phacoemulsification surgery and 83.48% for GATT only (26). In this study, the complete success rate of the GATT group was 63.0%, and the qualified success rate was 81.5%, which was approximately the same as that reported in a previous study.

Canaloplasty is a procedure whereby Schlemm’s canal is mechanically enlarged to improve aqueous outflow. The canaloplasty procedure can be performed via an ab externo surgical technique or via an ab interno surgical technique with a clear corneal incision. This technique aims to restore the physiological outflow pathways of the aqueous humor (27). The clinical outcomes and safety profile of ABiC have been investigated in several clinical studies. Koerber and Ondrejka (28) demonstrated that ab interno canaloplasty as a standalone procedure or combined with phacoemulsification using iTrack leads to a reduction in IOP and glaucoma medication use up to 24 months postoperatively. In 45 eyes with or without cataract surgery, Khaimi (29) reported a significant decrease (61%) in the mean number of medications taken at 36 months (1.89 ± 0.93 versus 0.60 ± 0.82), and 56% (14/25) of the eyes were medication free. Gillmann et al. (30) reported that the mean IOP decreased from 23.6 ± 7.4 mmHg preoperatively to 14.2 ± 2.9 mmHg (−39.8%; *p* < 0.001) after 12 months. The complete success rate was 50%, and the good success rate was 83.3%. Similar to our previous study, the 12-month cumulative mean IOP was 19.0 ± 5.2 mmHg, and the complete surgical success rate was 56% in the ABiC group (20).

Our study revealed a significant reduction in IOP and that results of GATT combined with ABiC are similar to those of previously published studies (11, 31, 32). We found that in our GATT + ABiC group, IOP decreased by 23.73% from baseline (24.5 ± 8.4 mmHg to 16.6 ± 2.3 mmHg) at 12 months postoperatively and to 15.5 ± 2.4 mmHg, with a 26.15% reduction from baseline, at 24 months postoperatively. The number of antiglaucoma medications used decreased from 3.7 ± 0.8 to 0.9 ± 1.3 at 12 months postoperatively and to 0.9 ± 1.1 at 24 months postoperatively. Our results were the same as those of Al Habash et al. (11), who reported the outcomes of GATT combined with ABiC in conjunction with phacoemulsification; the mean IOP was 13.30 ± 1.30 mmHg (IOP reduction of 32.7%), the number of antiglaucoma medications was reduced from 3.4 ± 0.6 (range: 2–4 medications) to 1.1 ± 1.0 (range: 0–2 medications) at 12 months postoperatively, and the overall success rate of GATT combined with ABiC in this study was 79.6%, which was similar to our qualified success rate of 76.9%. Terveen et al. (31) also described patients with OAG who underwent canaloplasty and trabeculotomy with the OMNI surgical system. The mean IOP before surgery was 22.3 (4.3) mmHg with 2.2 (1.3) medications, and at the last follow-up (mean 11 months), the IOP was 17.2 mmHg with 1.8 medications. Klabe et al. (32) reported the results of trabeculotomy/viscodilation for OAG 24 months after the procedure; the mean IOP decreased from 24.6 (3.0) mmHg to 14.9 mmHg, and the mean number of medications used was 0.5 (−1.4 medications) over a 24-month period. The overall success rate was 73%. Our results in the GATT combined with ABiC group showed comparable IOP lowering, antiglaucoma medication reduction and success rates to those of previous studies.

Previous studies have suggested that combining two different surgical procedures in the same setting addresses multiple concerns regarding outflow resistance and could, therefore, serve as an indirect indicator of surgical success (33). Overall, our study revealed that GATT + ABiC was not superior to GATT alone in controlling IOP in patients with OAG. The mean IOP, medication count and success rate were not significantly different between the two groups at either 12 or 24 months postoperatively. This means that there was no advantage associated with the combination treatment, as we hypothesized; however, the mechanism underlying the combined procedure requires further investigation. In our subgroup analysis, patients with uncontrolled baseline IOP (Subgroup A) benefit more from surgery, while medication-dependent patients (Subgroup B) show modest medication reduction but lower overall success. For patients with IOP > 21 mmHg, both procedures offer high success, and success rates are lower for medication-dependent patients (IOP < 21 mmHg on ≥3 meds), which need warranting cautious patient selection and closer monitoring.

Researchers believe that the application of viscoelastic material during microcatheter insertion during ABiC allows gentle stretching and separation of the compressed tissue of the trabecular meshwork and sclera and allows any herniated trabecular meshwork tissue to be withdrawn from collector channels (20); however, this effect is transient. The extreme traction force generated as the microcatheter is pulled out of the canal during GATT also stretches the outflow pathway. The trabecular meshwork used in the first ABiC procedures was viscoelastic, allowing extreme and ineffective traction force during GATT. Another explanation is that during GATT combined with ABiC, the microcatheter needs to be inserted twice, and the second insertion may damage the tissue.

The most commonly reported complications after GATT and ABiC are hyphema and IOP spikes (34). Hyphema is considered a sign of surgical success and usually resolves spontaneously in the early postoperative period (22). IOP spikes after surgery are the second most common complication and are associated with an increased likelihood of failure (35). The mechanism linking IOP spikes to failure remains uncertain (36).

In terms of complications, both GATT and GATT + ABiC were safe for patients with OAG. No severe complications were observed in this study, and trabecular meshwork/Schlemm’s canal-based MIGS procedures are among the safest forms of MIGS, with mostly self-limited and non-vision-threatening complications (37). Hyphema resolved spontaneously by the second week in most patients. Approximately half of the patients in the GATT group and one third of those in the GATT + ABiC group experienced an IOP spike in the first 2 weeks. IOP spikes in the GATT group were similar to those in previous reports, but IOP spikes in the GATT + ABiC group were more frequently observed in our study than in previous reports (11). This high detection rate may be due to intensive early postoperative follow-up. In previous studies, the postoperative use of corticosteroids was proposed to be the cause of IOP spikes (9, 36). Although the patients in our study received corticosteroids after surgery, the administration of such medications was not associated with IOP spikes, especially within the first few days after surgery. Shi et al. (38) reported that a longer duration of postoperative IOP spikes is a risk factor for failure, and severe cases are more likely to have longer durations of IOP spikes; thus, close monitoring of IOP spikes is needed (19). Only one patient experienced membrane detachment, which is not a common complication, and anterior chamber plasty was performed on day 25 after surgery.

Our study has several limitations. First, this study was retrospective; thus, prospective randomized controlled trials are needed to further clarify this issue. Second, we did not perform multiple analyses of risk factors because of the small sample size and the complex conditions of the patients. Additionally, the small sample size may also limit the statistical power to assess minor variation between these two surgical procedures. There may be potential for selection bias, as the decision to perform GATT or GATT combined with ABiC was determined solely by the discretion of the surgeon. Future studies need longer follow-up periods and larger numbers of patients to evaluate both procedures. The study is also limited by size and follow-up. With the goal of evaluating 2-year outcomes, we included only patients with > 12 months of follow-up.

To our knowledge, this retrospective study is the first to directly compare the outcomes of GATT alone with those of GATT combined with ABiC for OAG. According to the 24-month results of our research, with or without ABiC, GATT appears to be safe and effective in decreasing the IOP and the number of antiglaucoma medications used. For MIGS, whether to perform multiple combined procedures remains to be explored.

## Data Availability

The original contributions presented in the study are included in the article/Supplementary material, further inquiries can be directed to the corresponding authors.
