# Supplementary material for: Comparative effectiveness and safety of GATT with or without ABiC in patients with open-angle glaucoma
Source: Front Med (Lausanne). 2025 Aug 26;12:1581608. doi: 10.3389/fmed.2025.1581608 (PMC12418601; doi:10.3389/fmed.2025.1581608)
Supplement: Supplementary file 1 [file Table_1.docx]

**Supplementary Table S1. The success rates by preoperative subgroups.**

|  | **Preop Subgroup** | **Group** | **Eyes (n)** | **Complete Success** | **Qualified Success** | ***P* value** |
| --- | --- | --- | --- | --- | --- | --- |
| POM 12 | IOP > 21 mmHg | GATT | 12 | 83.3% (10/12) | 100% (12/12) | 0.243* |
|  |  | GATT+ABiC | 9 | 77.8% (7/9) | 88.9% (8/9) |  |
|  | IOP < 21 mmHg on ≥3 meds | GATT | 8 | 50.0% (4/8) | 75.0% (6/8) | 0.454* |
|  |  | GATT+ABiC | 9 | 44.4% (4/9) | 44.4% (4/9) |  |
| POM 24 | IOP > 21 mmHg | GATT | 12 | 83.3% (10/12) | 91.7% (11/12) | 0.632* |
|  |  | GATT+ABiC | 9 | 66.7% (6/9) | 77.8% (7/9) |  |
|  | IOP < 21 mmHg on ≥3 meds | GATT | 8 | 50.0% (4/8) | 62.5% (5/8) | 0.443* |
|  |  | GATT+ABiC | 9 | 33.3% (3/9) | 55.6% (5/9) |  |

^*^ Chi-square test
